# Dopamine Modulates the Excitability of Projection Neurons in the Robust Nucleus of the Arcopallium in Adult Zebra Finches

**DOI:** 10.1371/journal.pone.0082497

**Published:** 2013-12-05

**Authors:** Congshu Liao, Songhua Wang, Xuan Pan, Guoqiang Hou, Dongfeng Li

**Affiliations:** School of Life Science, South China Normal University, Key Laboratory of Ecology and Environmental Science in Higher Education of Guangdong Province, Guangzhou, Guangdong, P. R. China; University of Iowa, United States of America

## Abstract

**Background:**

The nervous system in songbirds is an accessible system for studying vocal learning and memory in vertebrates. In the song system, the anterior forebrain pathway (AFP) is essential for song learning and the vocal motor pathway (VMP) is necessary for song production. The premotor robust nucleus of the arcopallium (RA) located in the VMP receives input from the AFP. The RA receives dopaminergic innervations from the periaqueductal gray and ventral tegmental area–substantia nigra pars compacta, but the physiological functions of this projection remain unclear. In this study, we investigated the effects of dopamine (DA) on the excitability of projection neurons (PNs) in the RA.

**Methodology:**

We recorded the electrophysiological changes from neurons in brain slices of male adult zebra finches using a whole-cell recording technique.

**Conclusions/Significance:**

We found that DA significantly increased the excitability of RA PNs. Furthermore, a D1-like receptor agonist increased the excitability of RA PNs, and a D1-like receptor antagonist suppressed the excitability induced by DA. However, a D2-like receptor agonist had no effect on the excitability of RA PNs. Moreover, the D2-like receptor agonist did not change the excitability induced by the D1 receptor agonist. These findings suggest that DA can significantly increase the excitability of RA PNs and that D1 receptors play the main role in regulating the excitability of RA PNs in response to DA, thereby providing direct evidence toward understanding the mechanism of DA signal mediation by its receptors to modulate the excitability of RA PNs.

## Introduction

Birdsong is controlled by the song system. The song system is a network of discrete areas devoted to song learning and production [1], and consists of two functional pathways: the vocal motor pathway (VMP) required for song production and the anterior forebrain pathway (AFP) necessary for song learning, recognition, and plasticity [2–4]. The robust nucleus of the arcopallium (RA) receives neuron projections from both the posterior and anterior pathways of their respective neuron types. The RA is made up of two cell types, namely projection neurons (PNs) and interneurons [5]. RA PNs exhibit highly phasic bursts of action potentials (APs) during singing, and are similar to the pyramidal tract neurons of lower layer 5 of the mammalian motor cortex [6,7]. 

Dopamine (DA) is involved in highly motivated, goal-orientated, or anticipatory responses to stimuli associated with reward, including sexual behavior, feeding, and drugs of abuse [8–15]. In songbirds, DA receptors are mainly distributed in the Area X, RA, and high vocal center (HVC). The RA mainly receives dopaminergic (DAergic) inputs from the periaqueductal gray and ventral tegmental area (VTA) DAergic cell groups, and shows expression of D1-like and D2-like family receptors [16,17]. It was reported that the VTA plays a role in motivation and reward [18]. The number of tyrosine hydroxylase-immunoreactive neurons in the periaqueductal gray is correlated with courtship phenotypes [19]. In addition, D3 receptors belong to the D2-like family with higher expression in the RA PNs of birds. D3 receptors are located in VMP nuclei, suggesting their specialized role for vocal output [17]. These observations indicate that DA in the RA is important for song production, but the detailed mechanism remains unclear. Thus, the role of DA and its receptors in RA PNs has attracted our attention. 

In this study, we examined the effects of DA on the excitability of PNs in the RA. Our data showed that the excitability of RA PNs was increased by DA and a D1 receptor agonist. A D1 receptor antagonist blocked the excitability of RA PNs induced by DA, while a D2 agonist had no effects, suggesting a critical role of D1 receptors in regulating DA-mediated RA PN excitability. D1 receptors mainly affected the excitability of RA PNs in response to DA. Our results differ from the effects of DA in Area X of zebra finches, where activation of D1 receptors increases neuronal firing and activation of D2 receptors decreases neuronal firing [20]. However, our results are similar to the effects that were observed in the basolateral amygdala complex PNs [21] and dorsal striatum in rats, in which D1 receptors mainly mediated excitation of the neurons [22,23].

## Materials and Methods

### Slice preparation

All experiments were carried out in accordance with the university and national animal guidelines. The care and use of animals reported on in this study were approved by the Institutional Animal Care and Use Committee at South China Normal University and in accordance with National Institutes of Health guidelines (scnu20070033).

Twenty-seven adult male zebra finches (*Taeniopygia guttata*) (>90 days old) were obtained from a local breeder. The birds were housed in stainless steel cages (23.5×22.5×27.5 cm), and each cage contained a pair of male and female birds. Briefly, the birds were anesthetized with 10% chloral hydrate and then rapidly decapitated. The brains were dissected into ice-cold, oxygenated (95% O_2_ and 5% CO_2_) slice solution, consisting of (in mM) sucrose 248, KCl 5, NaHCO_3_ 28, glucose 10, MgSO_4_·7H_2_O 1.3, and NaH_2_PO_4_·H_2_O 1.26. Coronal brain slices (250 μm thick) containing the RA were cut with a vibrating microtome (MA752;WPI, Sarasota USA), collected in artificial cerebrospinal fluid (ACSF) that had been warmed to 37°C, and allowed to cool to room temperature. The slices were allowed to recover in a holding chamber for at least 1.5 h, and were equilibrated to room temperature before recordings were made. The standard ACSF consisted of (in mM) NaCl 125, NaHCO_3_ 25, NaH_2_PO_4_·H_2_O 1.27, KCl 2.5, MgSO_4_·7H_2_O 1.2, CaCl_2_ 2.0, and glucose 25, and was adjusted with sucrose to a final osmolarity of 350 mOs.

### Whole-cell recordings

For whole-cell recording, a slice was transferred to a submerged-type chamber, where it was continuously exposed to ACSF saturated with 95% O_2_ and 5% CO_2_ at room temperature (22–26°C) at a rate of 2.0 mL/min. The RA and its surrounding tissues were distinguishable under a BX51WI microscope connected to a DIC-IR video camera (Olympus, Tokyo, Japan). At high magnification (40×), the RA neurons were visualized. Recording pipettes were fabricated from borosilicate glass using a Flaming-Brown puller (Micropipette Puller P-97; Sutter Instrument Co., Novato, CA), and then filled with a pipette solution containing (in mM) KMeSO_4_ 120, NaCl 5, HEPES 10, EGTA 2, Mg-ATP 2, and Na_3_-GTP 0.3 (pH 7.2–7.4; 340 mOs). The electrode resistance was in the range of 4–7 MΩ. Tight-seal whole-cell recordings were obtained using standard techniques. The drugs used in the experiments included DA, S-(-)-SKF-38393 hydrochloride, (*R*)+(+)-SCH-23390 hydrochloride, (-)-quinpirole hydrochloride, (S)-(-)sulpiride, and tetrodotoxin (TTX) (Sigma-Aldrich, St. Louis, MO). All drugs were bath applied.

### Experimental protocols and analysis

We monitored the excitability by counting the number of APs evoked by a suprathreshold current pulse injection. The intensity of the current pulse, in the range of 50–200 pA with increments of 50 pA, was predetermined for each experiment to ensure that the number of spikes elicited was in the middle of the firing range before any drug application. The duration of a current pulse was 0.5 s. The interval between consecutive pulses was in the range of 60–90 s, and was regular throughout each experiment. 

We identified PNs in the RA based on three distinct intrinsic properties as follows: (1) regular APs that fired spontaneously; 2) slow time-dependent inward rectification of hyperpolarizing current pulses; and 3) regular firing of depolarizing current pulses. Non-projection neurons have no APs that fire spontaneously, no time-dependent inward rectification of hyperpolarizing current pulses, and irregular spiking in response to depolarizing current injection [5]. After identifying the neurons, we examined their responses to DA as described below. To characterize the effects of DA on the excitability of RA PNs, we employed the average number of spikes evoked by three current pulses before drug application as the measure of “pre-drug” excitability. We used the average number of spikes evoked by three pulses at 8 min after the onset of drug application (or two pulses around the end of the drug application if the drug application was <6 min) as the “during-drug” excitability. We took the average number of spikes evoked by three pulses at 8 min after the onset of washing as the “wash” excitability. The stimulated neuron-evoked firing rate was normalized by the pre-drug excitability. The membrane potential at the onset of an AP was taken as the spike threshold [20]. The spontaneous firing rate was calculated at the beginning of the recording as soon as it stabilized after patch rupture. We observed tonic spontaneous regular spiking at frequencies in the range of 0.5–10 Hz before drug application. 

### Data analysis and statistics

Clampfit 9.2 (Axon Instruments, USA) and OriginPro 8.0 (OriginLab, Northampton, MA) software programs were used for data analysis. APs were detected using the event detection package of Clampfit 9.2. Events with a peak amplitude of 50 mV or higher and a rising time of about 0.5 ms were detected automatically, and the results were analyzed with Origin Pro 8.0. Cells with a resting membrane potential that was more negative than −55 mV were used for the study. The series resistance, typically between 15 and 20 MΩ, was monitored during the recording, and cells with changes of >30% in the series resistance were not included in the analysis. 

Means were compared using paired two-tailed Student’s *t*-test or one-way ANOVA tests. Differences were considered to be significant for values of *p*<0.05. All numerical data are presented as the mean ± SD.

## Results

### DA has an effect on the excitability of RA PNs

**Figure 1:**
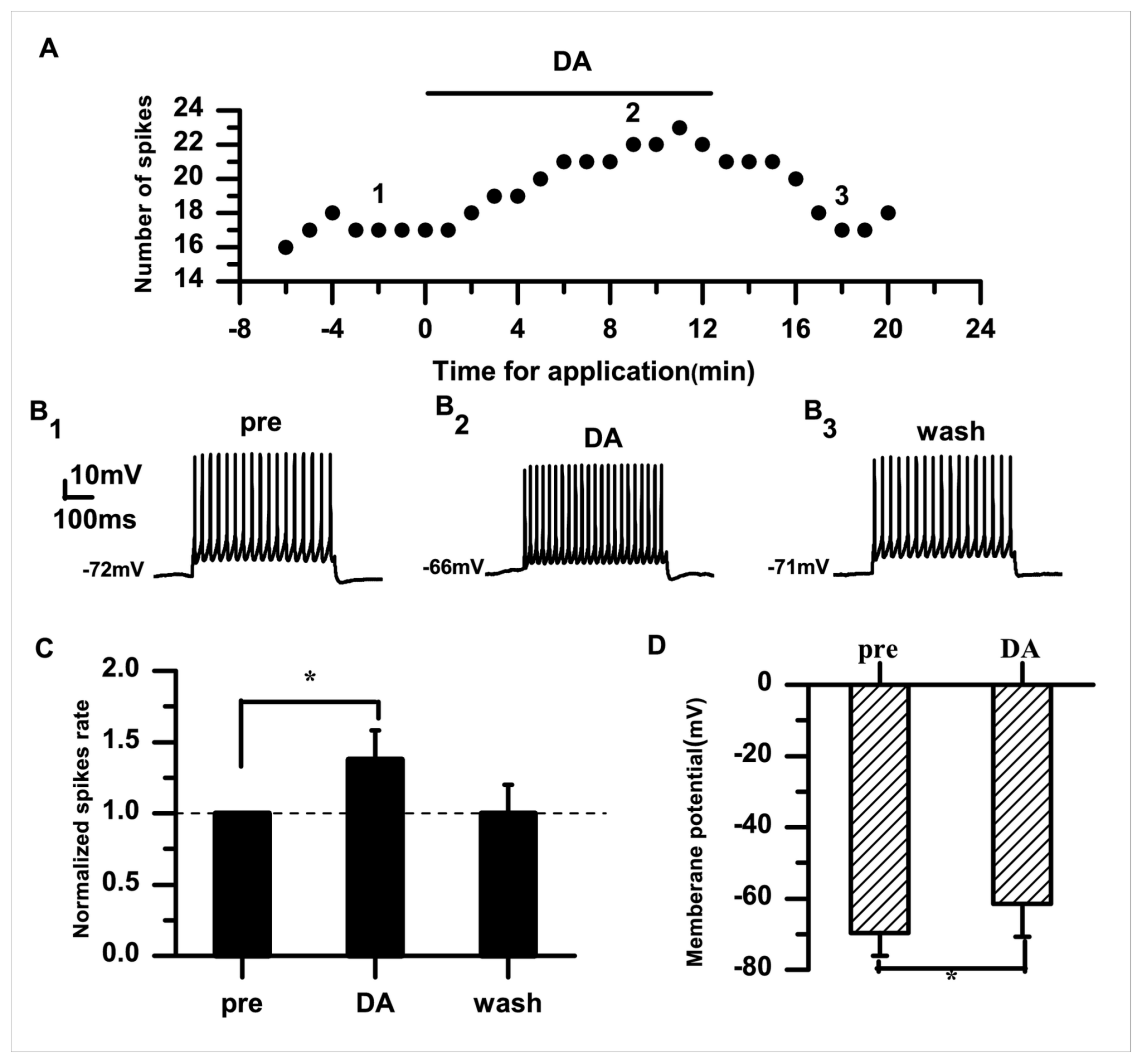
Effects of DA (50 μM) on the excitability of RA PNs. A. Numbers of spikes evoked by suprathreshold current pulses (100 pA; 500 ms). B_1–3_. Example traces from the experiment shown in (A). The numbers shown in (B) indicate the timing of each example trace. C. DA significantly increases the normalized spike rate in RA PNs. The dashed line indicates the control level (100%). D. Effects of DA on the membrane potential (*p<0.05).

**Figure 2:**
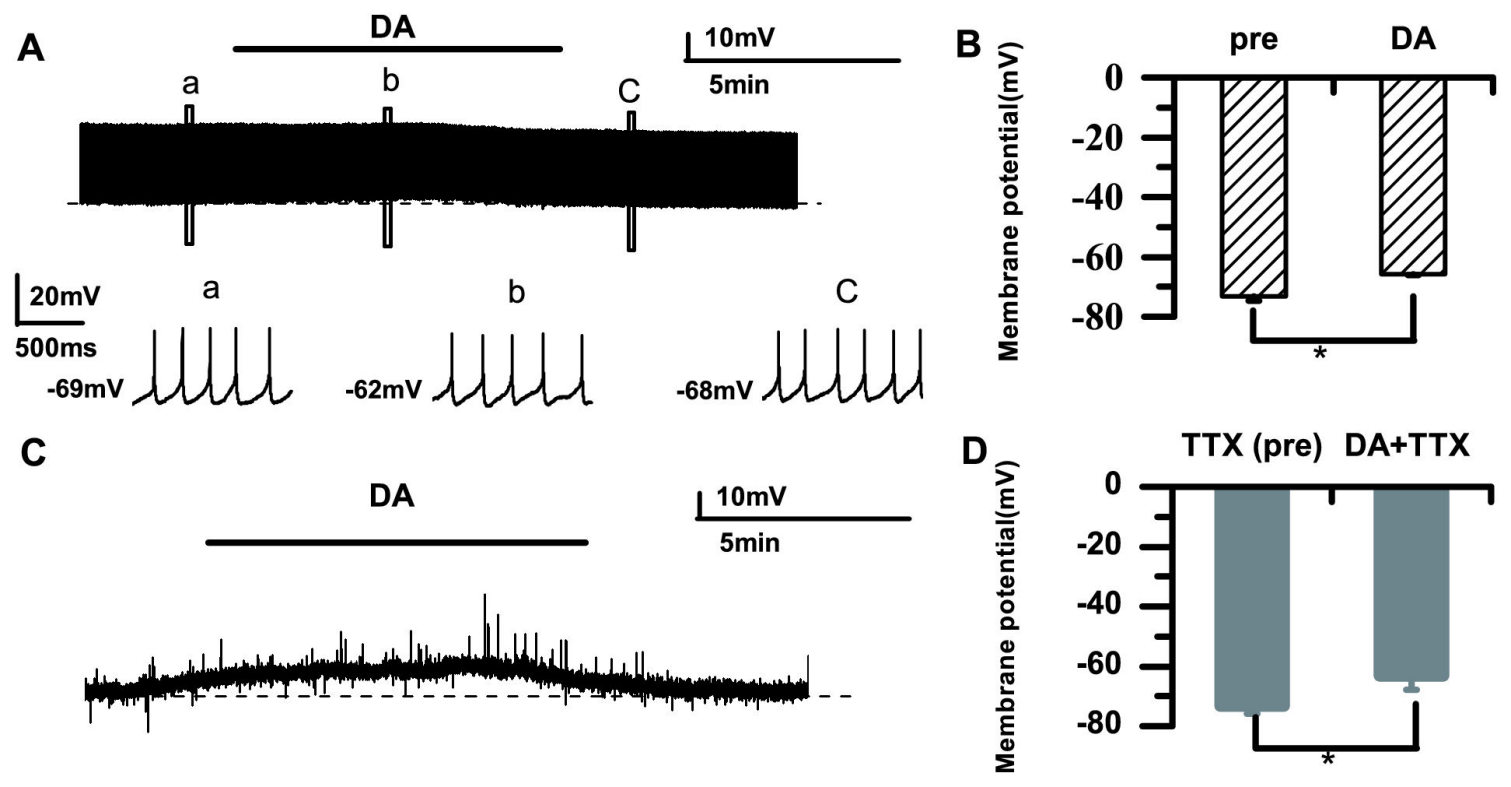
Membrane potential changes after DA application (50 μM). A. DA (50 μM) markedly increases the membrane potential of PNs, under conditions with no stimulation. a–c. Raw traces in the boxed areas showing the spike frequency before DA application (a), after DA application (b), and after washing (c). The dashed line represents the initial membrane potential. B. Average membrane potential changes without stimulation. C. DA produces depolarization. The dashed line represents the initial membrane potential. D. Average membrane potential changes induced by DA in the presence of TTX (1μM) (*p<0.05).

The excitability of RA PNs was reversibly enhanced by bath application of DA (50 μM). An example of the responses to a 100-pA current injection before and after DA application is shown in Figure **1A**. In this example, each 0.5-s current pulse elicited 17 spikes per pulse before DA application (Figure **1B** **_1_**). In the presence of DA, the numbers of evoked spikes slowly increased to 22 spikes per pulse during the same depolarizing stimulus after DA application for 10 min (Figure **1A, 1B** **_2_**). As shown in Figure **1C**, the normalized spike rate was significantly increased during DA application compared with the control (DA: 1.38±0.21; *p*<0.05; *n*=20) and returned to the baseline after washing. We also observed membrane potential depolarization (pre: −69.63±6.50 mV; DA: −61.45±9.30 mV; *p*<0.05; *n*=20) (Figure **1D**). The membrane potential may be affected by two main factors: 1) the injection current; and 2) the effect of the DA application. To examine whether electrical stimulation accounted for the cell depolarization, we recorded spontaneous AP firings before and after DA application. The example in Figure **2A** shows that DA caused membrane potential depolarization without stimulation. The average membrane potential changed from −73.21±1.43 mV to −65.91±0.23 mV (*p*<0.05; *n*=6) (Figure **2B**). Our data indicated that DA caused cell depolarization without any stimulation, suggesting that DA affects the excitability of RA PNs and modulates their membrane potential. Given that Na^+^ currents have a prominent role in membrane potential depolarization [24,25], we examined the effects of TTX on the membrane potential. In the presence of TTX, DA also caused membrane potential depolarization (pre: −73.32±2.57 mV; DA: −63.19±4.66 mV; *p*<0.05; *n*=6) (Figure **2C, 2D**). These results suggested that DA enhanced the excitability of RA PNs via activation of DA receptors, but not through Na^+^ currents.

### Effects of D1-like DA receptors on RA PNs

**Figure 3:**
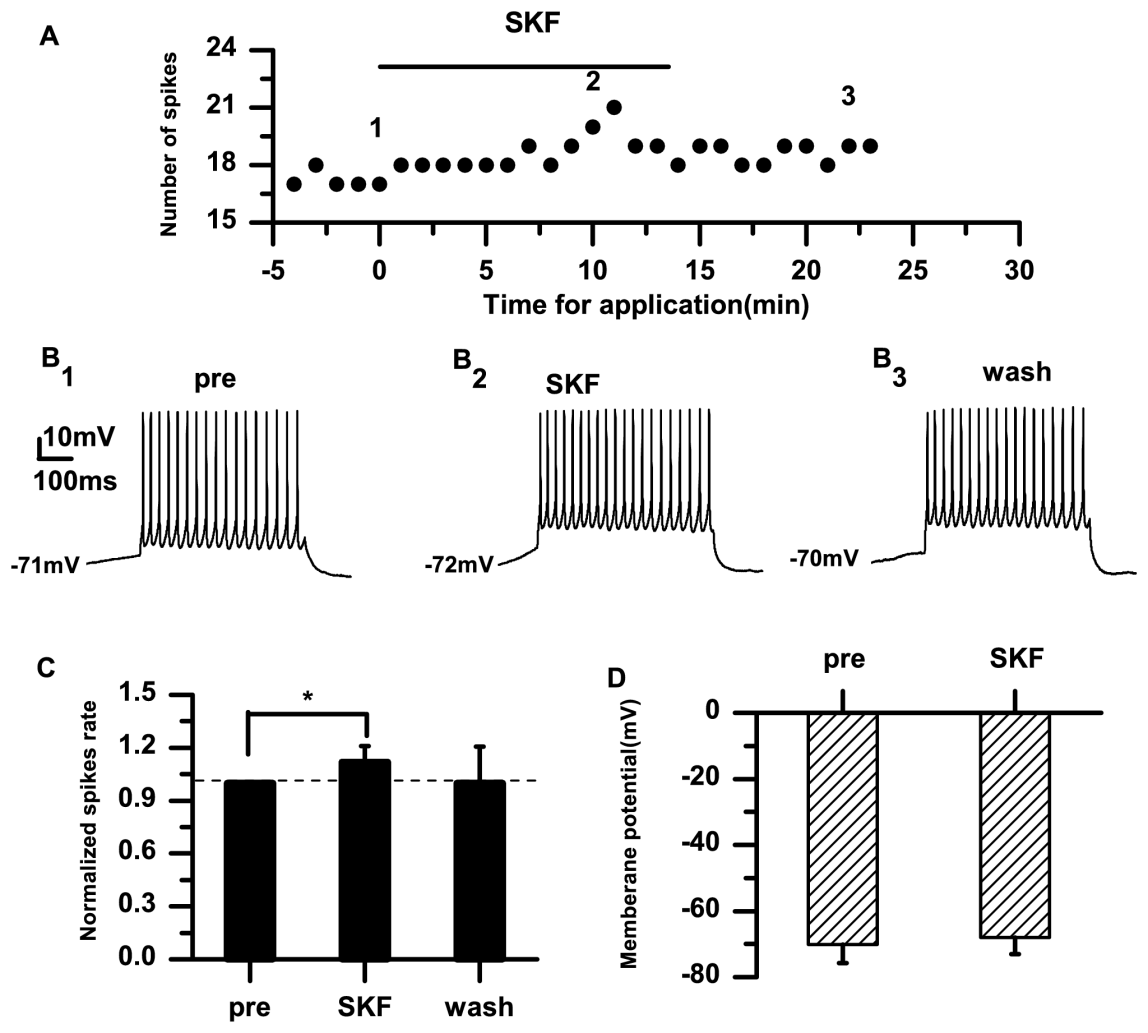
Effects of the D1-like receptor agonist SKF-38393 (10 μM) on the excitability of RA PNs. A. Numbers of spikes evoked by suprathreshold current pulses (100 pA; 500 ms). B_1–3_. Example traces from the experiment shown in (A). The numbers shown in (A) indicate the timing of each example trace. C. The D1-like receptor agonist SKF-38393 significantly increases the normalized spike rate in RA PNs. The dashed line indicates the control level (100%). D. Effects of SKF-38393 on the membrane potential (*p<0.05).

To further examine the effects of DA receptors in the RA, we applied the D1-like DA receptor agonist SKF-38393 (10 μM). The example in Figure **3A** shows that SKF-38393 increased the number of evoked spikes. In this example, each 0.5-s current pulse elicited 17 spikes per pulse before SKF-38393 application (Figure **3B** **_1_**). The cell elicited 21 spikes per pulse during the same depolarizing pulse in the presence of SKF-38393 for 8 min (Figure **3B** **_2_**). SKF-38393 significantly increased the normalized spike rate in RA PNs (SKF: 1.12±0.09; *p*<0.05; *n*=11) (Figure **3C**). However, the average membrane potential remained unchanged (pre: −70.10±5.67 mV; SKF: −67.93±5.03 mV; *p*=0.58; *n*=11) (Figure **3D**).

**Figure 4:**
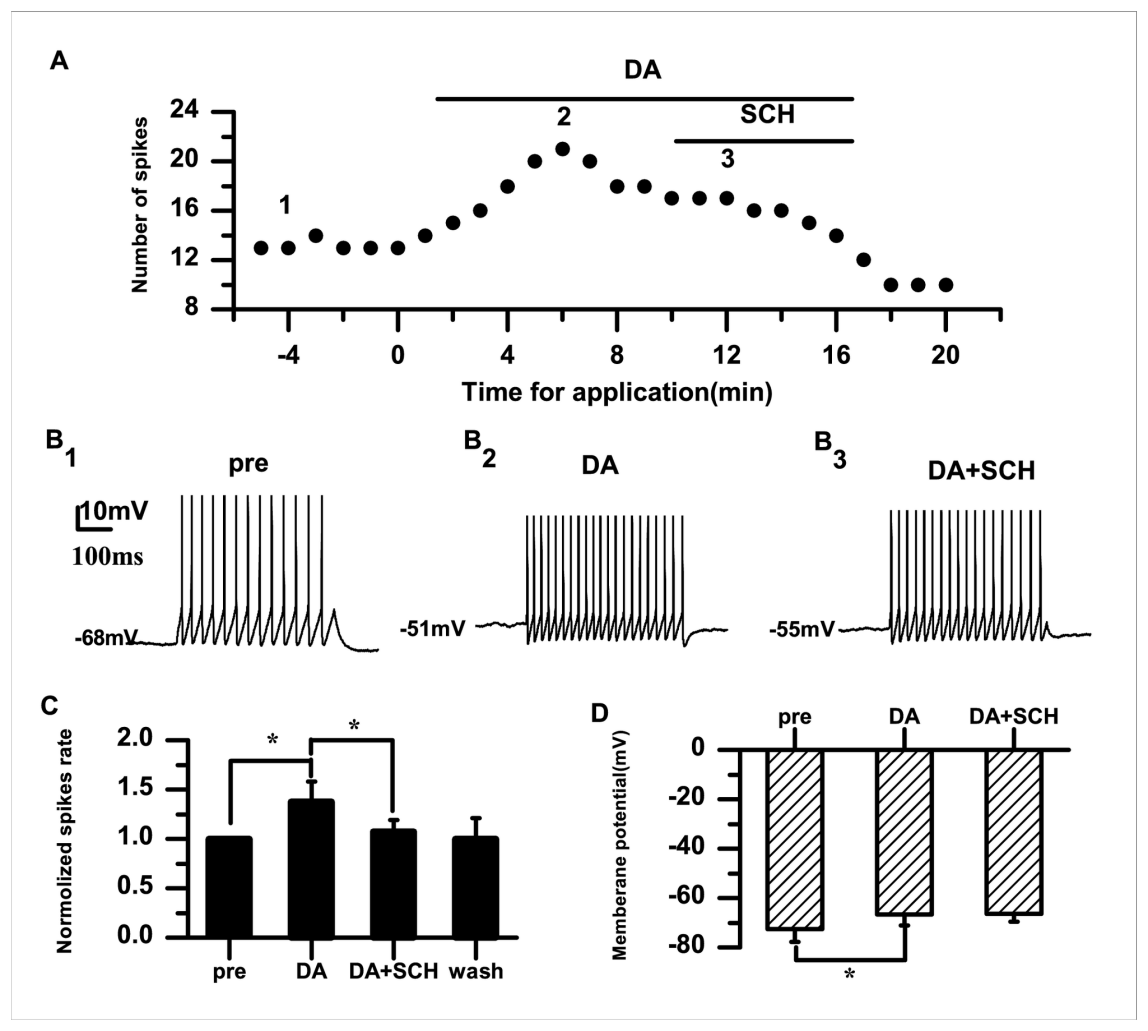
Effects of the D1-like receptor antagonist SCH-23390 (20 μM) on the excitability of RA PNs. A. In the presence of DA (50 μM), SCH-23390 (20 μM) inhibits the increase in evoked firing. B_1–3_. Example traces from the experiment shown in (A). The numbers shown in (A) indicate the timing of each example trace. C. The D1-like receptor antagonist SCH-23390 significantly decreases the normalized spike rate induced by DA. The dashed line indicates the control level (100%). D. Effects of DA and SCH-23390 on the membrane potential (*p<0.05).

To further examine whether D1 DA receptors contribute to the effect of DA, we applied DA and the D1 receptor blocker SCH-23390 (20 μM). The example in Figure **4A** shows that SCH-23390 reduced the DA-induced spiking activity. The normalized DA-induced spike rate was blocked by SCH-23390 (DA: 1.38±0.21; DA+SCH: 1.07±0.11; *p*<0.05; *n*=7) (Figure **4C**). We observed that membrane potential depolarization was induced by DA (pre: −72.63±5.06 mV; DA: −66.71±4.45 mV; *p*<0.05; *n*=7) (Figure **4D**). Consistent with the D1-like DA receptor agonist SKF-38393 results, the membrane potential remained unchanged after SCH-23390 application (DA: −66.71±4.45 mV; DA+SCH: −66.37±3.29 mV; *p*=0.99; *n*=7) (Figure **4D**). Collectively, these results indicated that DA caused membrane potential depolarization that was not mediated via D1 receptors, suggesting that D1 DA receptors are, at least, one of the main factors contributing to the enhancing effect of DA on the PN activity. 

### Effects of D2-like DA receptors on RA PNs

**Figure 5:**
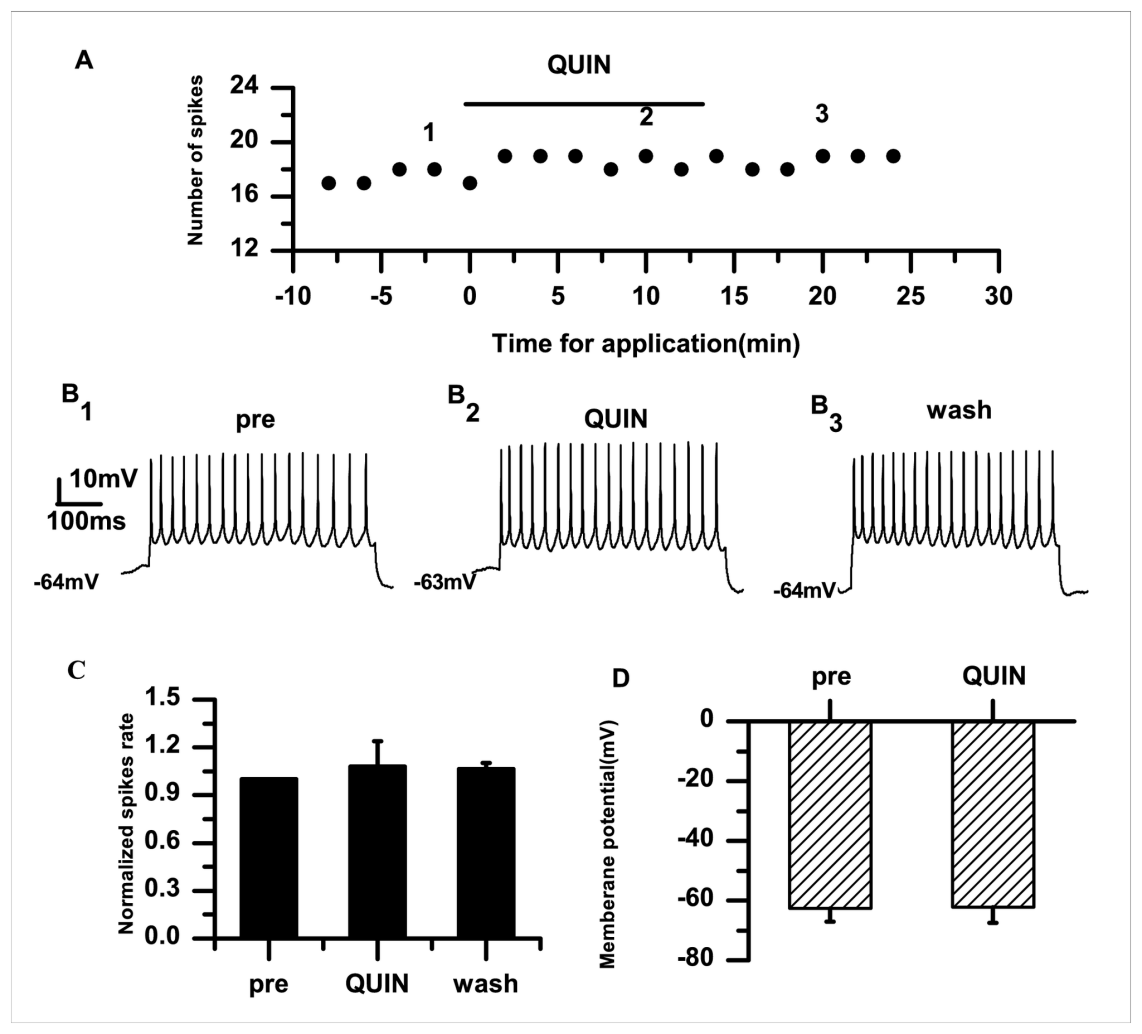
Effects of the D2-like receptor agonist quinpirole (10 μM). A. Application of quinpirole (10 μM) has no effect on the number of evoked firings. B_1–3_. Example traces from the experiment shown in (A). The numbers shown in (A) indicate the timing of each example trace. C. Quinpirole does not significantly change the normalized spike rate in PNs in the RA. The dashed line indicates the control level (100%). D. Effects of quinpirole on the membrane potential.

D3 receptor, which belongs to the D2 family, is highly expressed in the RA [17]. Therefore, we examined whether D2 receptors were involved in modulating the effects of DA. To this end, we applied the D2 DA receptor agonist quinpirole (10 μM). The example in Figure **5A** shows that quinpirole (10 μM) did not change the number of spikes after application for 5–6 min. Quinpirole had no effects on the normalized spike rate (quinpirole: 1.08±0.156; *p*=0.31; *n*=6) (Figure **5C**) or the membrane potential (pre: −62.56±4.48 mV; quinpirole: −62.20±5.35 mV; *p*=0.66; *n*=6) (Figure **5D**).

**Figure 6:**
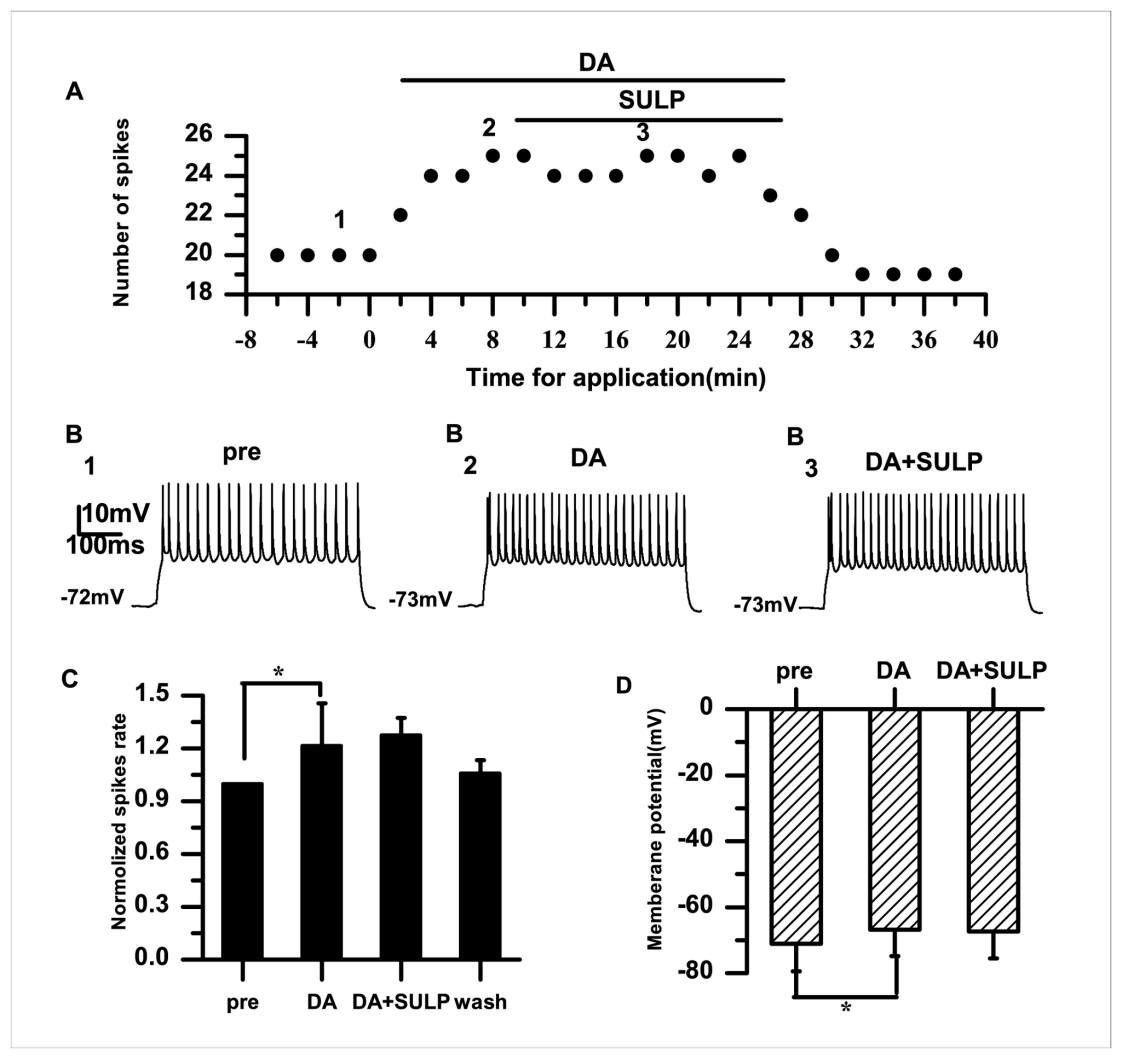
Effects of DA (50 μM) and sulpiride (10 μM) on the excitability of RA PNs. A. In the presence of DA (50 μM), sulpiride (10 μM) does not influence the evoked firing. B_1–3_. Example traces from the experiment shown in (A). The numbers shown in (A) indicate the timing of each example trace. C. The D2-like DA receptor antagonist sulpiride does not significantly change the normalized spike rate induced by DA. The dashed line indicates the control level (100%). D. Effects of DA and sulpiride on the membrane potential (*p<0.05).

To further examine the role of D2 receptors in the RA, DA and the D2 receptor blocker sulpiride (10 μM) were applied. Examples of the results are shown in Figure **6A and B**. When sulpiride (10 μM) was applied in the presence of DA, the normalized spike rate was 1.28±0.10, and did not differ significantly from that before sulpiride application (1.22±0.24; *p*=0.62; *n*=6) (Figure **6C**). In the presence of DA, membrane potential depolarization occurred (pre: −71.09±8.35 mV; DA: −66.80±8.01 mV; *p*<0.05; *n*=6) (Figure **6D**). However, we did not find any difference in the membrane potentials before and after sulpiride application (DA: −66.80±8.01 mV; DA+SULP: −67.36±8.18 mV; *p*=0.99; *n*=6) (Figure **6D**).

**Figure 7:**
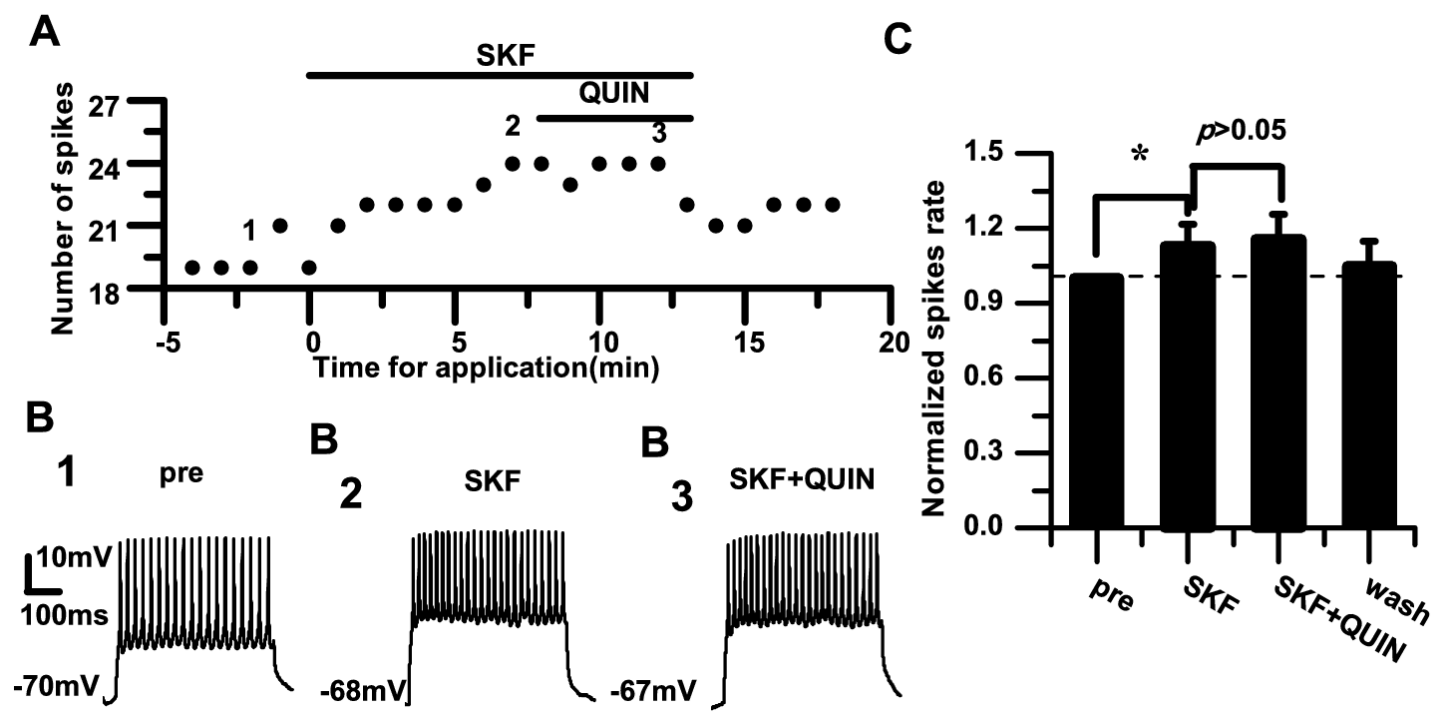
Effects of the D1-like and D2-like receptor agonists on the excitability of RA PNs. A. Effect of quinpirole (10 μM) application after SKF-38393 (10 μM) application. B_1–3_. Example traces from the experiment shown in (A). The numbers shown in (A) indicate the timing of each example trace. C. Effects of quinpirole application after SKF-38393 application. The dashed line indicates the control level (100%) (*p<0.05).

To further verify whether D2 receptors are involved in mediating the excitability of RA PNs, we applied the D1 and D2 receptor agonists. We found that the D2-like DA receptor agonist quinpirole (10 μM) had no effects on the evoked firing induced by SKF-38393 (10 μM) in PNs in the RA (Figure **7A**). Consistent with the above experiment, application with the D1 receptor agonist SKF-38393 significantly increased the normalized spike rate (*p*<0.05; *n*=6). However, the normalized spike rate showed no further change during D2 receptor agonist quinpirole application (SKF: 1.13±0.09; SKF+QUIN: 1.16±0.10; *p*=0.94; *n*=6) (Figure **7C**).

## Discussion

The data from the present study demonstrate that DA enhances the excitability of RA PNs, indicated by the increased spike rate and membrane potential depolarization (Figure **1**). Activation of D1 receptors also enhanced the excitability (Figure **3**). However, activation of D2 receptors did not significantly affect the excitability of RA PNs (Figure **5**). These results suggest that D1, not D2, receptors mainly mediate the effects of DA on the RA PN activity. This idea was further supported by our observations for the excitability of RA PNs during the application of D1 and D2 receptor agonists. In these analyses, the D2 receptor agonist had no additional effects on the excitability induced by the D1 receptor agonist (Figure **7**). D3 receptors are associated with cognitive functioning in both healthy individuals and those with neuropsychiatric disorders [26]. Although D3 dopamine receptors belonging to the D2 family are present at high levels in the RA PNs [17], quinpirole, which has a greater affinity for D3 receptors than for D2 receptors [27], did not cause observable effects on the D3 receptors in the RA PNs. There are two possible explanations for these findings. First, the D1 DA receptors or N-methyl-D-aspartate (NMDA) tone in the RA PNs may have been strong enough to mask the D3 receptor-mediated effects on the RA PNs directly. Second, in the RA, the differential DA receptor expressions could be cell-type specific, and D3 receptors may not be expressed in the PNs [17]. In addition, we found that D1 receptors only increased the spike rate, while neither SKF-38393 nor SCH-23390 was able to change the membrane potential (Figure **3**, Figure **4**). These results implied a positive effect of DA on the PN excitability mainly through D1 receptors. Certainly, the DA-induced neuron excitability may also involve other mechanism, due to the lack of membrane potential changes in the presence of the D1-like receptor agonist. 

DA receptors are involved in modulation of the adenylyl cyclase-protein kinase A pathway. D1 receptors were reported to be capable of elevating the level of cAMP and stimulating protein kinase A, thereby activating phosphorylation of DA and cAMP-regulated phosphoprotein (DARPP-32) [28]. The D2 receptor is negatively coupled to adenylyl cyclase and reduces the level of DARPP-32 phosphoprotein [28]. On the one hand, D1 receptors can activate the phosphorylation of DARPP-32, resulting in NMDA receptors maintaining their state of phosphorylation. The RA receives excitatory glutamatergic inputs from the HVC and LMAN(lateral magnocellular nucleus of the anterior nidopallium) that are mediated by a mixture of NMDA receptors [5]. On the other hand, Meitzen et al. [29] predicted that the RA would become more sensitive to the sparse HVC input with increased excitability when the RA PNs fire more rapidly under breeding conditions, or make them more likely to produce an AP in response to synaptic input. Combined with our results, we further speculate that DA mainly acts via D1 receptor-mediated effects on the signal transformation of excitatory inputs from the HVC to the RA PNs.

DA and DA receptors in the RA modulate different physiological functions in birds in a complex manner. Jarvis et al. [30] showed that the context-dependent gene (ZENK gene) expression is dramatically different in the RA. Hara et al. [31] reported that the ventral tegmental area–substantia nigra pars compacta (VTA-SNc) may not directly affect song motor output. Unilateral lesions of the VTA-SNc DAergic system caused bilateral effects on the downstream RA nuclei in different social contexts by modulating the egr-1 gene expression in lateral Area X of the striatum. These findings imply that DA modulates neural activity in the RA involving gene-level alterations and is social context dependent. The present study demonstrates that DA increases the excitability of RA PNs, which is mainly mediated by D1 receptors. We have provided direct evidence toward understanding of the mechanism by which DA receptors mediate DA signals to modulate the excitability of RA PNs, and given further insights into the role of DA in the RA.
